# Coatings Based on Essential Oils for Combating Antibiotic Resistance

**DOI:** 10.3390/antibiotics13070625

**Published:** 2024-07-04

**Authors:** Anita Ioana Visan, Irina Negut

**Affiliations:** National Institute for Lasers, Plasma and Radiation Physics, 409 Atomistilor Street, 077125 Magurele, Ilfov, Romania; anita.visan@inflpr.ro

**Keywords:** essential oils, coatings, natural antimicrobial, microbial resistance, bioactive compounds

## Abstract

In the current era of widespread antimicrobial resistance, the utilization of essential oils (EOs) derived from plants has emerged as a promising alternative in combating pathogens that have developed resistance to antibiotics. This review explores the therapeutic potential of essential oils as valuable tools in restoring the efficacy of antibiotics, highlighting their unique ability to affect bacteria in multiple ways and target various cellular systems. Despite the challenge of elucidating their precise mode of action, EOs have shown remarkable results in rigorous testing against a diverse range of bacteria. This review explores the multifaceted role of EOs in combating bacterial microorganisms, emphasizing their extraction methods, mechanisms of action, and comparative efficacy against synthetic antibiotics. Key findings underscore the unique strategies EOs deploy to counter bacteria, highlighting significant differences from conventional antibiotics. The review extends to advanced coating solutions for medical devices, exploring the integration of EO formulations into these coatings. Challenges in developing effective EO coatings are addressed, along with various innovative approaches for their implementation. An evaluation of these EO coatings reveals their potential as formidable alternatives to traditional antibacterial agents in medical device applications. This renaissance in exploring natural remedies emphasizes the need to combine traditional wisdom with modern scientific advancements to address the urgent need for effective antimicrobial solutions in the post-antibiotic era.

## 1. Introduction to the Topic and Justification of the Review Need

The rise in antimicrobial resistance has renewed interest in plant-based medicinal remedies, including essential oils (EOs).

It is important to find alternative solutions because the issue of antibiotic resistance is a pressing global healthcare crisis. The overuse and misuse of antibiotics have led to bacteria developing resistance, creating ‘superbugs’ that are challenging to treat. The pharmaceutical industry’s response has involved using broad-spectrum antibiotics and promoting their use in animal husbandry and crop cultivation, contributing to the problem. The illicit use of antibiotics, particularly counterfeit drugs in developing countries, further exacerbates the issue.

To address this challenge, it is crucial to promote responsible antibiotic use, invest in new drug development, and increase public awareness about antibiotic stewardship. Collaboration between healthcare professionals, researchers, policymakers, and the pharmaceutical industry is essential for a comprehensive solution.

Despite being overlooked by modern medicine’s advancements, EOs have shown promising results against various bacteria and offer a valuable tool against antibiotic resistance. Unlike antibiotics, EOs affect bacteria in multiple ways, enhancing their effectiveness in fighting infections when antibiotic options are decreasing. This underscores the need for alternative antimicrobial strategies and techniques to combat and prevent the effects of antibiotic resistance [1,2,3,4].

EOs are effective in treating infections and offer protection with a low risk of promoting resistance, making them a promising solution to antibiotic resistance due to their ability to target and neutralize airborne bacteria and viruses [5]. Preventing infections from nosocomial pathogens is crucial. Essential oils (EOs) show strong antimicrobial activity against various bacterial strains, including resistant ones, offering alternative treatment options and reducing dependence on conventional antibiotics. This broad efficacy can improve patient outcomes and healthcare delivery; thus, integrating EOs into infection control protocols and patient care can significantly transform healthcare practices. EOs can act as powerful air purifiers by inhibiting the growth of pathogenic fungi, thus ensuring a clean and healthy environment. Their volatility allows them to prevent the germination and growth of fungal spores, reducing the spread of infections. For example, tea tree oil effectively prevents *Aspergillus Niger* spore germination, thereby lowering the risk of fungal infections and promoting overall well-being.

There is an increasing need for antimicrobial coatings based on EOs as they offer a natural, effective solution for preventing microbial contamination and enhancing public health by reducing the spread of harmful pathogens on surfaces in various environments [6].

**Figure 1:**
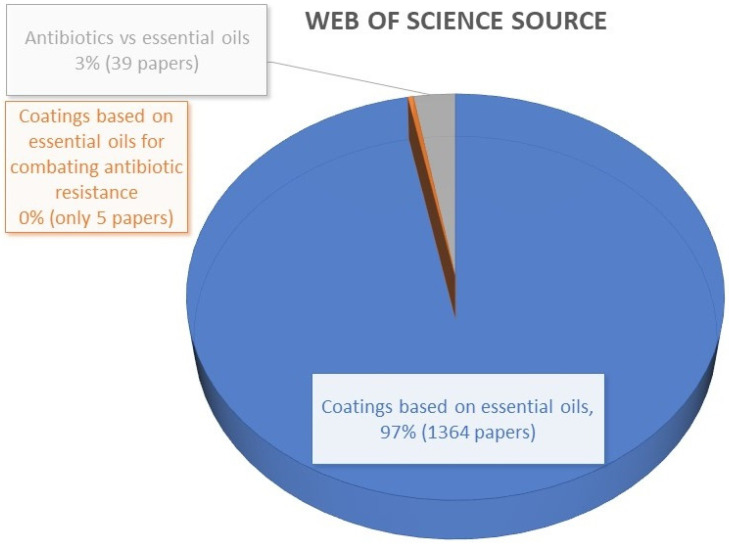
Justification of the review need.

A review on the topic of coatings based on EOs for combating antibiotic resistance is justified by their demonstrated efficacy against resistant bacteria and the urgent need for alternative antimicrobial strategies in the face of rising antibiotic resistance (Figure 1). The use of coatings based on EOspresents a promising approach to combating antibiotic resistance, a critical issue as a post-antibiotic era is approaching where even minor infections could become lethal. Thus, a comprehensive review of the use of EO-based coatings is justified to explore their dual role in enhancing the effects on both antibiotic-resistant and non-resistant bacteria and to identify factors that could optimize their antimicrobial activity. This exploration is not only timely but essential, given the severe morbidity and mortality rates associated with antibiotic-resistant infections and the urgent need for alternative strategies to combat these life-threatening challenges.

## 2. Chemical Composition of EOs

The myriads of functions displayed by EOs in plants originate from the large diversity of their chemical constituents. These substances can be small hydrocarbons (terpenes and sesquiterpenes) or other components of various degrees of hydrophobicity. These secondary metabolites belong to several families. Although RNA is the sole informational molecule determining the amino acid sequence of proteins in a cell, the diversity of secondary metabolites originates from the combination of a single intermediate molecule, -ocyclols [isopentenyl pyrophosphate (IPP) and dimethylallyl pyrophosphate (DMAPP)], which form isoprenoid intermediates: di-, sesqui-, tri-, or tetraterpenes. Their crystalline and volatile terpenic structure allows them to play a critical role in the protection of plants [7].

**Figure 2:**
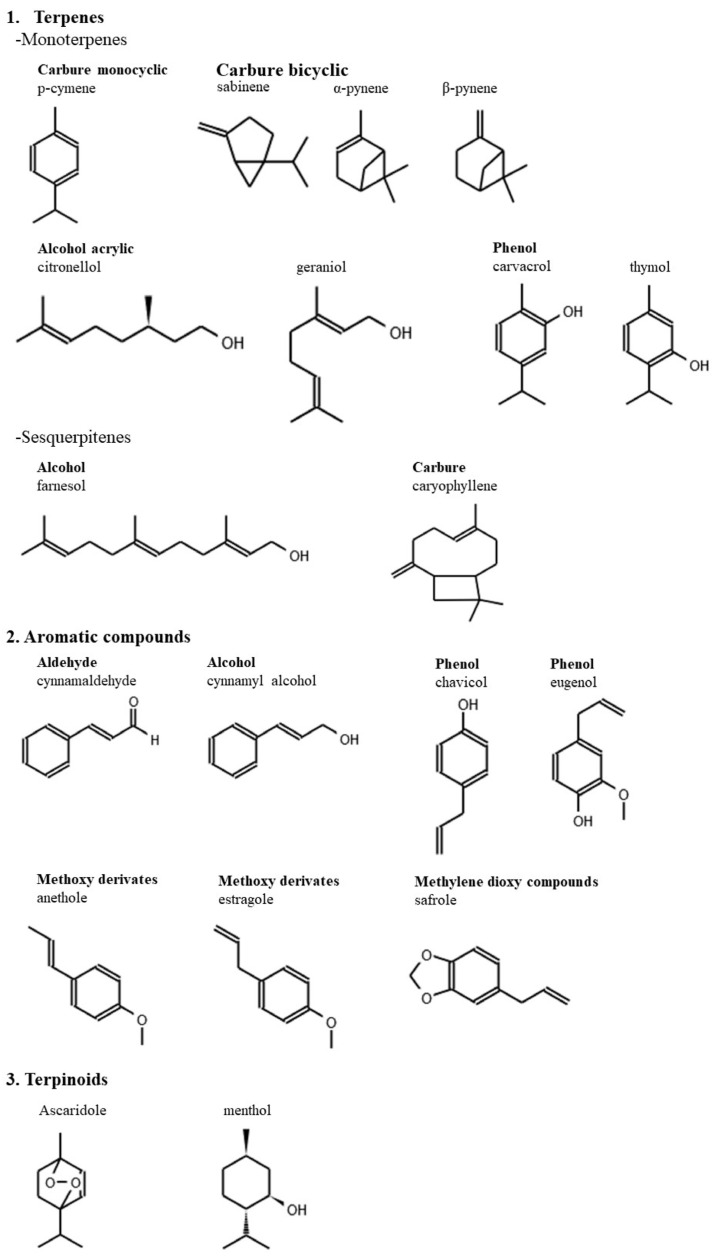
Some representative chemical structures of commonly found individual components of EOs [7].

EOs are complex volatile compounds, synthesized by plants via secondary metabolism [8,9,10]. These mixtures typically comprise 20–60 organic volatile components with molecular weights under 300, which often exist partially in vapor form due to their sufficiently high vapor pressures at ambient temperature and atmospheric pressure. Each EO is characterized by two or three dominant constituents, which are present in concentrations ranging from 20% to 70%. The primary active components of EOs generally include terpenes (such as monoterpenes and sesquiterpenes), aromatic compounds (including alcohols, phenols, methoxy derivatives, and aldehydes), and terpenoids (also known as isoprenoids). The components and aromatic qualities of EOs can be categorized into two main groups: terpene hydrocarbons and oxygenated molecules [11]. In fact, more than 9000 terpenoid structures are known. Terpenes can be either linear or cyclic. Depending on the number of carbon atoms and how they are connected, different chemicals are formed. For instance, terpenes contain ten carbon atoms, while sesquiterpenes contain 15 carbon atoms and diterpenes contain 20 carbon atoms (Figure 2). Moreover, the function of the isoprenoid biosynthetic pathway has been entirely described through research with various models, and trends in their evolution are well-understood. Plants, algae, fungi, and bacteria all produce isoprenoids. Consequently, terpenes were synthesized and have been able to interact with living matter for several billion years, influencing organization and engendering diversity among species. Additionally, some synthetic pathways rely on rapidly advancing oxygen chemistry. This enables precise selectivity towards desired molecules, presenting unprecedented opportunities [7].

(i) Terpenes and Terpenoids

Terpenes and terpenoids represent the most common classes of chemical compounds found in EOs. Terpenes, which are hydrocarbons, are synthesized when multiple isoprene units (C_5_H_8_) are linked together [12]. The synthesis of terpenes occurs in the cytoplasm of plant cells, beginning with acetyl-CoA and proceeding through the mevalonic acid pathway. Enzymes known as cyclases, which restructure the hydrocarbon backbone of terpenes, facilitate the formation of monocyclic or bicyclic terpenes. Terpene-specific synthetases then modify allylic prenyldiphosphate, forming the basic terpene skeleton. Following the synthesis of the IPP precursor, IPPs are sequentially added to generate the prenyldiphosphate precursor for various terpene classes. Ultimately, secondary enzymatic modifications, such as redox reactions, imbue the terpene skeletons with their distinctive functional properties [10]. The primary categories of terpenes in EOs are monoterpenes (C_10_H_16_) and sesquiterpenes (C_15_H_24_), although terpenes can also form longer chains such as diterpenes (C_20_H_32_) and triterpenes (C_30_H_40_). EO components that exhibit antimicrobial properties include p-cymene, limonene, menthol, eugenol, anethole, estragole, geraniol, thymol, γ-terpinene, and cinnamonyl alcohol [13]. Plants known for containing these antimicrobial agents encompass a diverse range, including angelica, bergamot, lemongrass, mandarin, mint, caraway, celery, citronella, coriander, eucalyptus, geranium, petitgrain, pine, juniper, lavandin, lavender, lemon, orange, peppermint, rosemary, sage, and thyme. These plants are valued not only for their aromatic qualities but also for their potential in therapeutic applications due to their antimicrobial constituents [14].

Terpenoids are synthesized by biochemically modifying terpenes, a process facilitated by enzymes that transfer or remove methyl groups and incorporate oxygen molecules. These modifications lead to various subgroups of terpenoids, including alcohols, phenols, esters, aldehydes, ethers, ketones, and epoxides, with examples such as thymol, carvacrol, linalool, linalyl acetate, citronellal, piperitone, menthol, and geraniol [15]. Several carotenoids support several functions in addition to protection against photo-oxygenation present in photosynthetic plants; they are crucial during fruit/flower ripening, photosynthesis, and protection against abiotic stresses such as photoinhibition provoked by excess light or high/low temperature. The level of terpenoids in carotid bodies is enhanced in response to hypoxia in rats, leading to the assumption that terpenes may be involved in oxygen sensing. Finally, bacteria and mitochondria also synthesize terpenoids, with most relying on a single alternative pathway that leads to the synthesis of precursor geranyl pyrophosphate (GPP) and geranylgeranyl pyrophosphate (GGPP). Cholesterol and dolichol in mitochondria and menaquinone in bacteria are synthesized through this process [16,17].

The sequestration of terpenoids plays a defensive role for plants as they can protect themselves from herbivory. Another important role is that terpenoids can contribute to plant survival in case of attack by being synthesized and released from the wound site, thus attracting the enemies (be they herbivores or herbivores’ natural predators). Recently, studies have demonstrated that terpenoids such as GPP, cystopterol acetate, and jasmonic acid have a positive correlation with induced defensive responses against herbivores. Oriental fruit flies generate high levels of induced terpenes, supporting their existence across different origins of terpenes and the conserved role of the compounds in plant–herbivore interactions [18].

Several terpenoids, especially monoterpenes, have a significant number of variations, such as cymene, carveol, limonene, myrcene, myristicin, or pinene. Many plants express monoterpene synthases (TPSs) involved in the biosynthesis of these monoterpenes. Moreover, plants synthesize other terpenoids from the GPP. Diterpenes can be divided into several classes, including cembratrienes, casbene, grayanene, stemodene, taxadiene, and labdanes, which are derived from GGPP. Diterpene synthase (diTPS) enzymes derived from copalyl pyrophosphate (CPP) and syn-copalyl pyrophosphate (sCPP) can produce several of these compounds. Additionally, the triterpenes that are derived from squalene are obtained from chrysanthemyl pyrophosphate (CPP), 8-hydroxy-CPP, and presqualene pyrophosphate (PSPP). Furthermore, sesquiterpenes can be classified as part of the linear carotenoid family, cyclic carotenoids (xanthophylls), and the larger cyclic sesquiterpenes, which include the irregular group of abietane and the clerodane diterpenoids [18].

Terpenoids are a large and diverse class of secondary metabolites, derived mainly from (IPP) and dimethylallyl pyrophosphate (DMAPP), which are synthesized via the mevalonate (MVA) and the 2-C-methyl-D-erythritol-4-phosphate (MEP) pathways. Terpenoids are known to play critical roles in key cellular processes and, due to their large structural diversity, can be used for different chemical and biological applications [19].

(ii) Phenylpropanoids

Phenylpropanoids, another class of aromatic compounds derived from the phenyl-propane structure, are produced via the shikimic acid pathway, leading to phenylalanine. This group includes phenols (e.g., eugenol and chavicol), aldehydes (e.g., cinnamaldehyde), alcohols (e.g., cinnamic alcohol), methylenedioxy compounds (e.g., apiole, myristicin, and safrole), and methoxy derivatives (e.g., anethole, estragole, and methyleugenols) [11].

Selected phenolic glycosides and a concentration of sugar in leaves exert a decoy action, being involved in insect-resistant responses. In the photosynthetic region of the leaves and stems of plants, phenylpropanoid cinnamic acid is transformed to form p-coumaric acid, which transforms in accordance with the biodiversity characteristics of the microbial flora formed in the phyllosphere. Many of the phenolic cinnamic derivatives have antibacterial activities against human pathogenic bacteria. However, sensitive bacteria that prevent the adverse effects of phenolic cinnamic derivatives have developed different detoxification pathways [20].

The naturally pure derivative of phenylpropanoid cinnamic acid can be transformed to form thousands and thousands of secondary phenolic products produced in nature. Aromatic and medicinal herbs exhibit a strong genotype nutrient response, hence altering essential oil aromatic profiles in accordance with environmental growing conditions rather than large metabolic changes in yield and growth rate. The shoots of aromatic plants transmit a wide variety of natural phenolic products, where it is believed that essential oils act as their primary chemical messengers. Both phenylalanine ammonia lyase and 4-cinnamate-CoA ligase are extensively expressed in the leaf chlorenchyma layer and the green part of leaves, which function as the sites of synthesis for the majority of the supply transpiring in persistent, low-volatility, partly glycolytic leaf oil types [21,22].

(iii) Alcohols, aldehydes, and ketones

Aldehydes, containing the CHO functional group, are important for flavor and fragrance, particularly in tropical fruit oils. Coniferyl aldehyde, multimethoxy dimethanol, and other aldehydes are all important in the biosynthesis of plant lignin, acting similarly to toxicity-generating reactive oxygen species (ROS) damage. At the same time, aldehydes such as benzaldehyde and anthranilic acid can be used as food additives to inhibit microbial growth, and ketal groups, such as cryptomeridiol, can be extracted from fruits. Furthermore, the *Pseudomonas fluorescens* reaction throws a protective carrot-like glycoprotein that plays a positive role in the anti-cancer and anti-inflammatory aspects of dry fruit analysis. The bacterial mechanism suggests that desirable properties can be used to improve nutritional and health functions. Meanwhile, plant aldehyde groups, such as vanillin and piperonal, show remarkable antioxidant properties and anti-proliferative potential in various cancer cell lines, which is an improvement over the commercially available BHT and α-T. As a group with good biological activity, vanillin, everthisobenzaldehyde, benzaldehyde, and other benzene derivatives, as well as alkadienals and thenal, have been investigated by the FDA due to potential contaminants from e-cigarettes and oral tobacco products that may present health risks—promoting tumor growth or worsening existing ones, affecting the immune system, and potentially affecting metabolism in systems [23].

Alcohols, typically containing a hydroxyl group, are generally known for targeting the plasma membrane, providing their antimicrobial activity. Cinnamyl alcohol, occurring in essential oils from the bark, leaf, and fruit, contains a phenylpropene structural skeleton with an allyl substituent, which is an inhibitor of lactic dehydrogenase from the oral pathogen Streptococcus gordonii. Other compounds, such as the benzene derivatives cinnamon alcohol, cumin alcohol, and phthalate cousin geraniol, which contain an OH group, have also been found to induce antimicrobial activity by altering the structure of bacterial membranes. Halitoxins, including compounds such as *E*-phenylmethyl-3-(l-methylpropyl)acryloxymethylphosphocholine (CI-583) and (*Z*)-3-dodecen-1-ol, are strong modulators of the enzyme DNA topoisomerase I, thus enhancing the inhibitory effects on NA synthesis, a mechanism also linked to the pepper compound elemecin. Sugar alcohol derivatives, such as derrifordioside F and erythritol, can be extracted from various plants and provide new sources for anti-MDR and antiparasitic agents [24].

## 3. Biochemical Properties of EOs

For terpenoids, cyclooxygenase, terpenoid precursor pools, terpenoid action assays, an enzyme essential for eicosanoid synthesis, and sterol biosynthesis have been identified as target systems. Proteins involved in these pathways have thus evolved specific binding cavities that often consist of both hydrophobic surfaces and tightly packed hydrophilic residues that interact directly with the ligand molecules. Genes encoding these enzymes are promising targets for drug development as hosts with altered genes may not be able to modify the EOs in a way that offers refuge from the host defense. Another protein target of terpenoids is the sterol biosynthesis enzyme. The timely depletion of geranylgeranyl diphosphate results in the collapse of the infection structure before the breach takes place. The essential oil can induce the specific protein-dependent signaling required for adaptive stress responses [25].

In spite of recent advances in the field, relatively little is known about the mechanism of action of many EO components, especially terpenoids. This is despite them being important economically, for example in food preservation, but also in anti-infective and other pharmacological treatments. This uncertainty is in large part due to the complex nature of volatile oils, which typically comprise tens of different components. Furthermore, the usual perception of EO components as hydrophobic molecules that are volatile at room temperature precludes the type of interactions considered to mediate other molecular actions. In addition, these hydrophobic molecules, when in an aqueous medium in vivo, may specifically interact with a few selected protein targets with high specificity to mediate desired pharmacological or antiseptic actions [26].

### 3.1. Antimicrobial Activity

The antimicrobial activity of EOs has been widely known and utilized for centuries due to their potential in combatting microbial infections. Their effectiveness has become increasingly important as microbes continue to develop resistance against antibiotics. Extensive evidence suggests that EOs possess remarkable activity against a wide range of pathogens, including fungi, viruses, and bacteria. Notably, certain EOs have the ability to completely eradicate all tested samples of specific bacteria, while a significant proportion demonstrates effectiveness against various types of microbes. This versatility is a distinct advantage compared to many antibiotics, which are often limited to fighting either Gram-positive or Gram-negative bacteria [27].

The ability of a given aromatic plant to inhibit or kill microorganisms is based solely on the properties of its EOs. The effective control of the various types of microorganisms (bacteria, fungi, and viruses) by aromatic molecules depends on physicochemical parameters, mainly the oxidation rate of the precursors of these extraordinarily reactive molecules, and their diffusion capacity within the medium. The mechanisms of their lethal effect on the vital structures of microorganisms clearly show that the engagement of these molecules mainly depends on physicochemical and morphological factors, and not on targeted biochemical routes. The biochemistry of microorganisms and that of aromatic molecules could explain the microbiological activity of these molecules, and therefore help to predict and significantly optimize their use [11,28].

Many EOs can disrupt bacterial cell membranes, leading to cell leakage and death. For instance, tea tree oil and eucalyptus oil are known to damage bacterial cell walls. Another example is *Melaleuca Alternafolia* oil, which has proven its efficacy against numerous antibiotic-resistant bacteria. Additionally, preparations derived from *Lamiaceae* spp have shown to be superior antimicrobials when compared to many commercially available pharmaceuticals [25].

Some EOs, such as oregano oil, can inhibit bacterial efflux pumps, which are mechanisms bacteria use to expel antimicrobial agents, thereby making them more susceptible to treatment. Bacteria often form protective biofilms that make them more resistant to antibiotics. EOs from cinnamon and thyme can penetrate and disrupt these biofilms, enhancing antibacterial effectiveness. EOs from clove and rosemary contain antioxidants which can reduce oxidative stress in bacteria, impairing their function and viability. When combined with antibiotics, certain EOs can enhance the effectiveness of these drugs, helping to overcome antibiotic resistance. This is seen with EOs from lavender and peppermint. Many EOs have broad-spectrum antibacterial properties, meaning they are effective against a wide range of bacterial strains, both Gram-positive and Gram-negative. For example, lemon and lemongrass oils have demonstrated effectiveness against various bacterial pathogens. The volatile nature of EOs allows them to penetrate deep into tissues and surfaces, ensuring comprehensive antibacterial action even in hard-to-reach areas [11].

Although multiple studies have established the impact of EOs on bacteria, the exact mechanisms by which they exert their antimicrobial effects remain largely undocumented. Various theories have been proposed, including the inhibition of microbial cell wall synthesis, enzyme inactivation, alterations in the microbial membrane surface through disruption of the phospholipid bilayer, and the prevention of respiration, resulting in damage to the cell’s genetic material. It is important to note that their action is likely complex and dependent on factors such the oil’s chemical composition and concentration, as well as the specific microbe being targeted, as mentioned by de O. Filho et al. [10,29]

One notable advantage of certain EOs over antibiotics lies in their ability to reverse or prevent the development of resistance exhibited by certain microbes towards standard drugs. This remarkable attribute further highlights the immense potential of EOs as vital tools in combating microbial infections and finding alternative therapies. With the increasing resistance of microbes against antibiotics, the urgent need for effective solutions has never been more apparent. EOs offer a promising avenue for exploration and continued research in the fight against microbial infections. Investigations into the antimicrobial properties of essential oils have had a significant impact on the medical field. By utilizing the potency of these natural substances, scientists and researchers have been able to create new methods for treating microbial infections [28].

One aspect of EOs is their ability to completely eliminate specific bacteria strains during testing. Moreover, they also demonstrate effectiveness against other types of microbes. This versatility sets EOs apart from many traditional antibiotics, which often have limited effectiveness against certain types of bacteria. This remarkable attribute further underlines their vast potential as essential tools in combating microbial infections and developing alternative therapies.

### 3.2. Anti-Inflammatory Effects

The most promising results with EOs are obtained by their application in acute and chronic skin diseases. Psoriasis, neurodermatitis, and atopic eczema show significant improvement after external use of creams or oils containing essential oils. Essential oils are also liable to exert an immunosuppressive effect; one research study showed that T-lymphocyte proliferation was strongly inhibited by certain essential oils. Care should therefore be taken when using these oils when some immunostimulation is wanted, such as in fatigue, convalescence, or in the elderly. However, recent clinical research suggested that the regular use of tea tree oil may have a role in the prevention of recurrent colds. The inhalation of EOs, as part of aromatherapy, has a whole series of suggestive names: epigenetic, psychophysiological, neuroneuroendocrinology, psychoimmunoendocrinology, neuroendocrinoimmunology, and neuroendocrino-immunomodulation all describe the action of essential oils through our sense of smell. The treatment of infections with essential oils can be considered a form of aromatherapy [30].

The most prominent effect of essential oils on inflammation is inhibition by the activities of COX (cyclooxygenase-1 and 2) and LOX (lipoxygenase). Inhibition by COX-1 (good pain) or by COX-2 (bad inflammation) and by LOX is essential to obtain an excellent anti-inflammatory action using essential oils. Not all EOs from the same species modulate these enzymes in the same way, especially if there are many different chemotypes in the EOs. However, chemotypes that contain a high content of phenylpropanoids and monoterpenes are excellent anti-inflammatory agents. Moreover, some essential oils have shown an excellent selectivity profile in their in vitro inhibition of COX activity. The selectivity index of several essential oils has been calculated and is indicative of a therapeutic window higher than that of established non-steroidal anti-inflammatory (ibuprofen) and selective COX-2 inhibitor (celecoxib and rofecoxib) drugs. These observations could mean that anti-inflammatory EOs are good candidates for drug development, with very few side effects. There are also important differences between various study models about the in vivo activity of selected EOs, so the application of further experiments in these models would appear to be warranted [31].

### 3.3. Antioxidant Properties

The hypothesis that many genes exist includes hereditary pathologies. Most lipophilic antioxidants that accompany oxidative damage are vitamin E and carotenoids, which are isoprenoid derivatives. It can be said that lipid-soluble chemical groups linked to phenolic groups can protect the cells from oxidative stress. Furthermore, the key relationship is that many polyphenols could act as antioxidants, intercept interconnected networks of ROS, and suppress mutagenesis and other alterations to cell growth. Such damage to proteins, lipids, and DNA can cause accelerated carcinogenesis. Essential oils exhibit antioxidant properties, indicating that an intake of certain EOs could help protect the body from oxidative stress [32].

ROS such as OH, O_2_^−^, H_2_O_2_, and ROO- are permanently produced in living organisms. Aerobic organisms possess superoxide dismutases (SODs), catalases (CATs), and ascorbate peroxidases (APXs), as well as glutathione peroxidases (GPXs), which prevent damage to the cell by detoxifying these ROS. ROS are induced by exogenous agents, a so-called oxidative stress that occurs in both animals and plants. In human beings, it is toxic agents that are responsible for inducing oxidative stress, such as heavy metals and drugs. The hypothesis that many ROS are induced by exogenous agents, so-called oxidative stress that genetically exists, includes hereditary pathologies [33].

The antioxidant activity of EOs plays a crucial role in augmenting their antimicrobial effects through multiple mechanisms. EOs contain a variety of antioxidant compounds, such as phenols, terpenes, and flavonoids, which possess the ability to scavenge ROS and mitigate oxidative stress within microbial cells [11]. By neutralizing ROS, EOs disrupt essential cellular processes and weaken the defense mechanisms of pathogens, rendering them more susceptible to antimicrobial agents [34]. Additionally, the antioxidant properties of EOs contribute to their ability to protect against oxidative damage and maintain cellular integrity, thus enhancing their overall antimicrobial efficacy [11]. Furthermore, the synergistic interactions between the antioxidant and antimicrobial components of EOs can potentiate their therapeutic effects and overcome microbial resistance mechanisms [34]. Therefore, understanding the antioxidant activity of EOs is essential for elucidating their antimicrobial effects and exploring their potential as natural alternatives to conventional antibiotics.

## 4. Methods of EO Extraction

The European Pharmacopoeia characterizes EOs as aromatic products typically derived from a botanically identified plant raw material through steam distillation, dry distillation, or a suitable mechanical method without the application of heat [35]. As stipulated by the European Pharmacopoeia, the extraction of EOs primarily involves a physical process from the aqueous phase that preserves its chemical integrity. EOs, which are volatile, clear, and occasionally colored in their liquid state, play a critical defensive role in plants by emitting strong flavors that deter herbivores. Chemically, EOs are soluble in organic solvents and are less dense than water [36]. They are synthesized from various plant parts, including buds, flowers, leaves, stems, twigs, seeds, fruits, roots, wood, and bark, and are stored in specialized structures such as glandular trichomes, secretory cells, cavities, and canals [11]. In the plant kingdom, only ~10% of species—amounting to over 17,000 species—produce EOs, hence qualifying as aromatic plants. The predominant families contributing to the global distribution of these aromatic plants include [36].

EOs are colored, fragrant, and complex hydrophobic liquids [37], also referred to as volatile oils [38]. These oils are characterized as secondary metabolites produced by aromatic plants [39] and are located in various plant parts, including flowers, roots, bark, stems, leaves, and seeds [40]. Due to their robust therapeutic properties, such as antibacterial, antiseptic, and antioxidant activities, EOs are effective in reducing antimicrobial resistance [41]^.^ Consequently, EOs derived from medicinal plants have been explored as potent antimicrobial agents within animal production systems [42]. The blend of compounds within oils gives them unique properties and fragrances, which some experts speculate provide the plants with an arsenal of defense mechanisms against bacteria, fungi, insects, and even herbivorous mammals.

**Figure 3:**
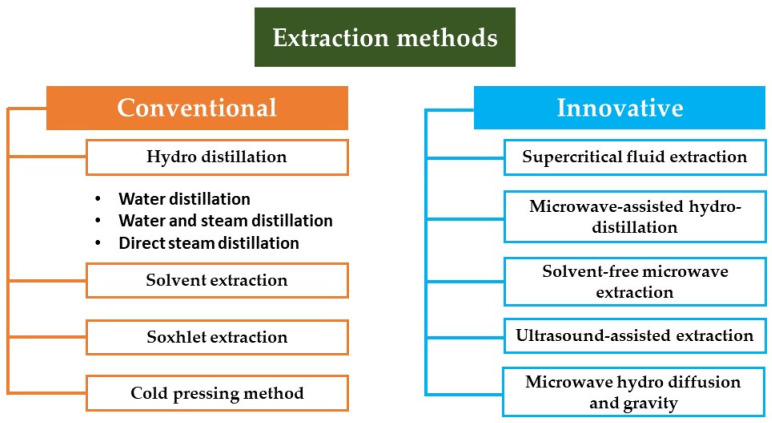
Essential oil extraction methods.

Extracting these oils from plants requires specific techniques (Figure 3) that are designed to preserve their aromatic and chemical properties, each with their own advantages and disadvantages (Table 1).

## 5. Mechanisms to Combat Bacterial Microorganisms

The bioactive compounds in EOs, such as terpenes, phenols, aldehydes, and alcohols [11], interact with microbial targets through various mechanisms, including disruption of cell membranes, inhibition of enzyme activity, and interference with microbial cell signaling pathways [10].

### 5.1. Mechanisms of Action at the Cellular Level

The study of EO cellular mechanisms of action reveals the intricate ways these natural compounds interact with cellular structures and processes, offering insights into their medicinal properties. It is essential to expand our research from microorganisms to their host, enhancing their functions in a supportive environment. Attention should focus on commensal organisms within our body, particularly the immune system, which benefits from microbial detoxification and hormonal support. Additionally, cultivating specialized microbiota could optimize host benefits [52,53].

**Chart 1:**
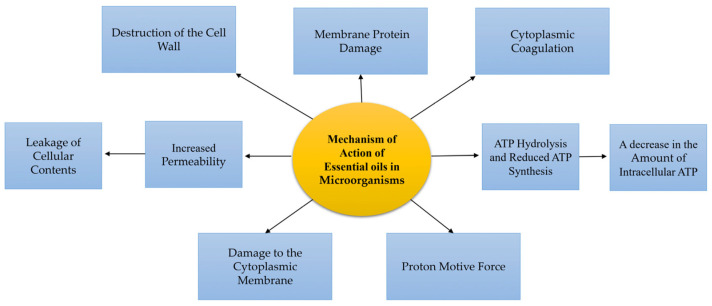
Various types of mechanisms of the activities of EOs on microorganisms [53].

This relatively simple and clear scheme portrays EOs as a concentrated essence of plant chemical defenses, efficiently disrupting the essential functions of the cells of pathogens (Chart 1).

The assumed oil resistance of many microbial pathogens, as well as a myriad of mechanisms exhibited by cells enabling them to withstand the bactericidal effects of EOs, was the focus of many papers in recent years. The pervasive resistant phenotype of cutaneous and systemic fungal pathogens could be attributed to growth rate-dependent pace-making skills and the slow but steady pace of qualitative improvement. They encounter a constant stream of endo-metabolites, but the pool of toxic exo-metabolites is relatively narrow, mainly represented by EOs, and only capable of eliminating the smallest, and thus most numerous, catabolism-denying microbes, thus delaying the moment when the used-up, hesitant, recalcitrant cells monopolizing the substrate supply can finally gather the go-ahead signal [54].

Broadly speaking, cellular targets of EOs can be divided into two major categories. Oxygenated monoterpenes mainly target mitochondrial respiration, deplete ATP levels, and induce a harmful chain of events that ends in apoptosis. Hydrocarbons appear to target inner motion and intracellular communication. Joining the metabolic superfluousness of the cell with augmented selflessness are two beautiful adaptations of essential oils, allowing cells to step aside when their usefulness is exhausted [55].

### 5.2. Enzyme Inhibition and Modulation

Enzyme inhibition possesses several connotations incorporating the action of an essential oil against microorganisms and insects that reside in a bioenergetic mode. For instance, the inhibition of unique enzymes or enzyme classes present solely in the causal microorganism, as well as enzyme inhibition that does not solely reduce the available energy supply and nutrient yield for the invading pest or pathogen in comparison to the host, leads to an abundance of metabolites that could possess signaling roles during microorganism growth and during infections. Importantly, enzyme inhibition as an antiviral and cell protective mechanism is also applicable. Although most published evidence appears in the phytochemical and pharmaceutical literature, experimental data demonstrate that essential oils have broad inhibitory activities towards enzymes and possess some structural preferences for enzyme families. The broad range of the published literature on the subject indicates the large potential of essential oils as both a lead and future aid in therapeutic designs against a variety of different enzyme-mediated diseases [56].

EOs exhibit antimicrobial activities largely due to their phenolic compounds, as noted by Fukuyama et al. [57].

In other examples, phenolic compounds such as thymol and carvacrol found in thyme and oregano oils have been shown to disrupt bacterial cell membranes, leading to leakage of cellular contents and ultimately cell death [58]. Additionally, terpenoids such as menthol and limonene can interfere with microbial enzymatic processes, disrupting essential cellular functions [59]. Furthermore, EOs may modulate microbial gene expression and signaling pathways, impacting vital cellular processes such as cell division and metabolism. Overall, the multifaceted antimicrobial effects of EOs result from the intricate interactions between their chemical constituents and microbial targets, highlighting their potential as natural alternatives to conventional antimicrobial agents.

The qualitative and quantitative compositions of EOs exhibit considerable variability [60]. This variability is influenced by several factors, which can be categorized into two main groups: intrinsic factors, which encompass the plant species, its developmental stage, and its interactions with the environment, such as soil type and climatic conditions; and extrinsic factors, which relate to the extraction methods used. Additional elements such as seasonal variations, specific plant organs, plant maturity, geographic origin, and genetic factors also play significant roles. Due to their complex interrelationships and combined effects, these factors often present challenges in distinguishing their individual contributions to the variability in essential oils [61].

**Figure 4:**
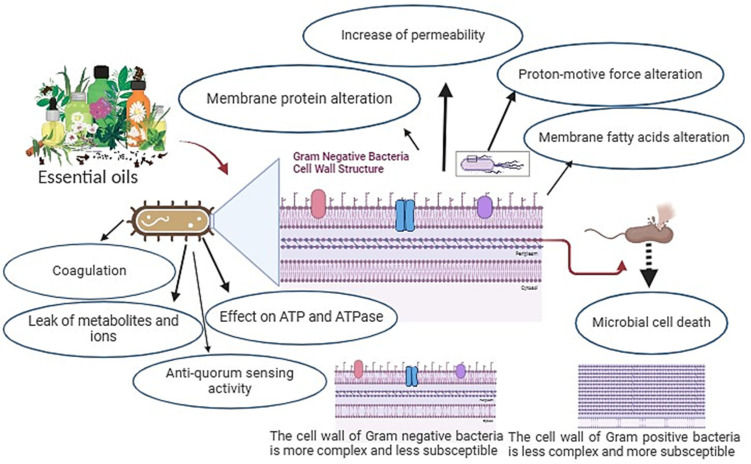
Mechanisms of EO actions leading to microbial cell death [62].

Figure 4 illustrates the mechanisms of EO action that culminate in microbial cell death, highlighting the various pathways through which these natural compounds exert their antimicrobial effects.

One of the best-known antimicrobial oils is tea tree oil (*Melaleuca alternifolia*), which is derived from the leaves of a plant that is indigenous to Australia. Recognized for its potent antiseptic properties, recent research has highlighted its effectiveness against a range of bacteria, including antibiotic-resistant strains. Tea tree oil disrupts bacterial growth, biofilm formation, and bacterial cell membranes. It shows efficacy against bacteria such as *Staphylococcus aureus*, *Escherichia coli*, *Streptococcus pyogenes*, and *Pseudomonas aeruginosa*. Its ability to penetrate biofilms makes it a potential alternative therapy for complex infections, with ongoing studies exploring its applications in wound healing, dandruff treatment, and dental care [62,63].

Another oil that has been widely researched and studied is bergamot oil (*Citrus aurantium var. bergamia*). This highly sought-after oil is extracted from the fragrant peel of the fruit, making it a valuable ingredient in both aromatherapy and premium-quality toiletries. Bergamot oil has been found to possess antibacterial effects that have garnered significant attention. In a comprehensive study, it was found that bergamot oil exhibited an impressive ability to completely eliminate *Streptococcus mutans* from saliva cultures. This discovery highlights the effectiveness of this oil in eradicating harmful bacteria. In addition, the oil also demonstrated significant efficacy in inhibiting the adherence of these bacteria to culture plates. This inhibition is highly crucial as it directly impacts the virulence of these organisms. *Streptococcus mutans*, a bacterium responsible for tooth decay, has sparked a significant interest in the utilization of EOs for improving oral health. The connection between *Streptococcus mutans* and the utilization of EOs has not only ignited significant interest but has also sparked an insatiable curiosity among professionals dedicated to improving oral health [64].

The ever-growing threat of bacterial strains developing resistance to multiple antibiotics has created a sense of urgency in exploring alternative antimicrobials [65,66]. EOs offer hope in combating bacteria that are not responsive to traditional antibiotics. They are now seen as crucial weapons against evolving bacteria due to the need to address antibiotic resistance [67].

## 6. EOs vs. Antibiotics

**Figure 5:**
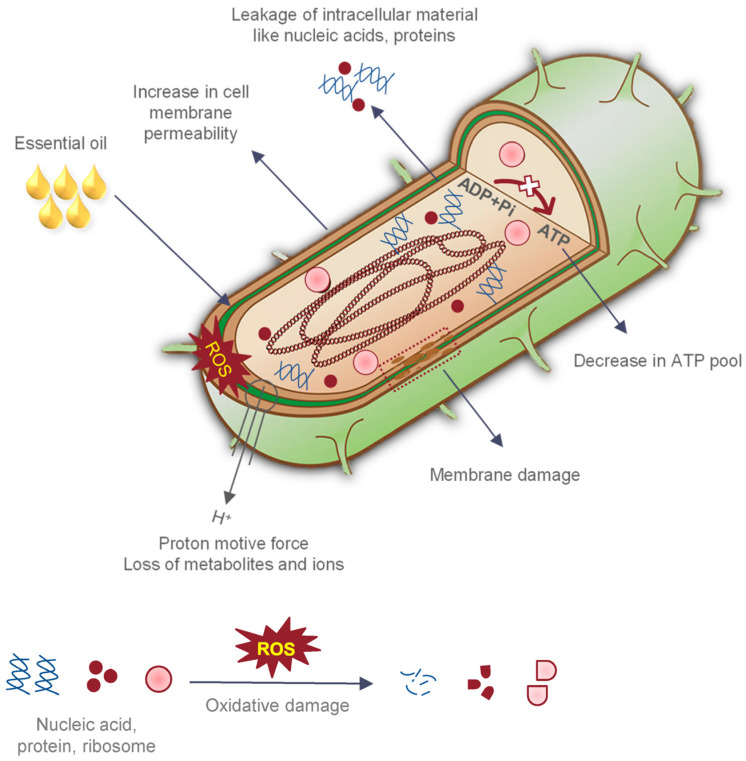
Mechanism of EOs vs. synthetic antibiotics [10].

Having explored the mechanisms by which EOs induce microbial cell death, this section compares essential oils with conventional antibiotics to evaluate their relative efficacy and potential advantages. As EOs have gained widespread recognition and acceptance as a highly potent and efficient alternative therapy for combating the ever-increasing issue of antibiotic resistance, it is only natural and justifiable to draw insightful comparisons between their extraordinary antimicrobial properties and the more conventional approach of conventional antibiotics. Figure 5 presents a comparative analysis of the mechanisms by which essential oils and synthetic antibiotics act against microbial cells, illustrating their distinct pathways and effects.

EOs and conventional antibiotics both exhibit antimicrobial properties, but they differ in their mechanisms and scope of efficacy. In Table 2, we have summarized a comparative analysis of EOs and conventional antibiotics.

In Table 3, some key findings regarding the studies on EOs vs. synthetic antibiotics can be found.

EOs and conventional antibiotics differ significantly in their origins and characteristics. EOs, derived from diverse plant sources, have a complex and variable composition of bioactive compounds that contribute to their antimicrobial properties. These compounds vary depending on plant species, geographic location, climate, soil conditions, and extraction methods. In contrast, conventional antibiotics are synthetically formulated and target specific bacterial functions or structures. The diversity and complexity of EOs add to their potential as versatile antimicrobial agents against a range of pathogens [76]. These variables introduce a multitude of possibilities for discovering and harnessing the antimicrobial power of EOs.

In contrast, conventional antibiotics, although highly effective in many cases, are limited by their narrow spectrum of activity and the increasing development of resistance among pathogenic microorganisms. This limitation arises because of the specific targeting mechanism of conventional antibiotics, which often target a single molecular target or pathway. EOs present a promising alternative to antibiotics due to their complex compositions that target multiple molecular pathways in microorganisms, making it difficult for resistance to develop. The synergistic interactions between compounds in EOs enhance their antimicrobial efficacy. EOs also offer additional therapeutic benefits such as antioxidant, anti-inflammatory, and immunomodulatory effects. Despite their potential in alternative medicine, the use of EOs is still constrained by various challenges and limitations.

Standardization and quality control of EO products remain essential to ensuring consistent composition and potency, as variations in these factors can significantly affect their therapeutic efficacy [77]. Furthermore, the safety and proper usage of EOs require careful consideration, as some oils may cause adverse reactions or interactions with certain medications. In conclusion, the utilization of EOs as an alternative therapy for combating antibiotic resistance is a fascinating and rapidly evolving area of research. Continued studies and advancements in the understanding of the complex mechanisms underlying the antimicrobial activity of EOs can pave the way for the development of novel treatments and strategies in the fight against microbial infections [78].

Recent research highlights that conventional antibiotics, despite their targeted approach, may not always completely eradicate bacteria. Emerging studies emphasize the potential of EOs in addressing microbial threats. EOs, known initially for their broad-spectrum effects, have demonstrated effectiveness against specific types of bacteria, underscoring their significant impact in combating infections [79]. EOs have been extensively studied and proven effective in inhibiting microbial growth, highlighting their potent natural defense capabilities. What sets them apart is their unique ability to selectively target and inhibit specific mechanisms crucial for the survival and reproduction of infectious microorganisms. This characteristic not only underscores but also enhances their potential as a novel approach to combating bacterial infections, marking a promising advancement in medical research, treatment, and microbial defense [80].

An example is *Melaleuca alternifolia* oil [81], which has been shown to inhibit a wide array of microbial growths. It accomplishes this by specifically targeting the synthesis of the cell wall of the organism. This is comparable to conventional antibiotics, which also target the bacterial cell wall in some cases. For example, penicillin inhibits the synthesis of peptidoglycan in the cell wall. However, such specific antibiotics are rendered ineffective against microbes whose resistance has rendered the target site invulnerable. Moreover, in addition to targeting the microbial cell wall, the oil of *Melaleuca alternifolia* exhibits promising effects on disrupting microbial membranes, leading to cell lysis and inhibition of microbial activity. Furthermore, research has shown that *Melaleuca alternifolia* oil not only acts on Gram-positive bacteria but also displays efficacy against Gram-negative bacteria and even some multidrug-resistant strains. Another significant advantage of *Melaleuca alternifolia* oil is its ability to penetrate the biofilms produced by certain microbes, making it an extremely potent weapon against persistent and challenging infections. These biofilms provide a highly protective environment for bacteria, allowing them to evade the immune system and develop resistance against traditional antibiotic treatments. However, *Melaleuca alternifolia* oil disrupts biofilms by effectively inhibiting their formation and weakening their structural integrity. This dual action of targeting both the cell wall and biofilms sets *Melaleuca alternifolia* oil apart from traditional antibiotics, making it an immensely promising alternative or adjunct therapy in the battle against microbial infections [82].

In terms of cost-effectiveness, EOs may present a more economical option compared to conventional antibiotics, particularly in resource-limited settings where access to pharmaceutical drugs is limited [83].

By incorporating and integrating the vast and diverse range of benefits, advantages, and merits of EOs, individuals can proficiently and competently combat and overcome these indomitable, unwavering, persistent, and stubborn infections, thereby ensuring and guaranteeing a comprehensive and all-encompassing approach, perspective, and outlook to health, well-being, and overall wellness [84].

## 7. Advanced Coating Solutions for Medical Devices

Following our comparison of EOs and synthetic antibiotics, this section will explore advanced coating solutions for medical devices and examine how these innovative technologies can enhance antimicrobial protection. Advancements in medical devices have given rare insights into whether the source of nosocomial infections is the result of an in situ foreign body or the handling processes during surgery and post-surgery wound management. Extra emphasis has been placed on the microbial colonization of non-living surfaces such as catheters, orthopedic fixation pins and joints, and various prosthetics. Bacterial adherence and biofilm formation on these devices have been widely recognized as the most prevalent cause of infections related to medical implants. It is for this reason that antibiotics have been impregnated into certain materials for controlled release. Unfortunately, the antibiotic cannot differentiate between the prosthesis or pin and the surrounding tissue. Furthermore, an inappropriate dosage compared to systemic antibiotics can lead to antibiotic resistance in the bacteria [85].

Extensive research has explored the potential of essential oils (EOs) for medical applications. Two pioneering studies have specifically investigated coating medical devices with EOs to significantly reduce bacteria and prevent infections, suggesting a promising approach to combating nosocomial infections [86]. The use of EOs for coating purposes allows for a targeted approach, directly focusing on the area where the device comes into contact with the body. This targeted delivery system ensures maximal effectiveness while minimizing the potential for systemic side effects. In addition to their ability to combat bacterial infections, EOs have also been shown to possess anti-inflammatory and immunomodulatory properties. These multifaceted characteristics make them even more appealing for use in medical device coatings. By reducing inflammation and modulating the immune response, EOs create an environment that discourages bacterial growth and colonization, thereby preventing infections and promoting faster healing. EOs can also be used alongside antibiotics to enhance their effectiveness against established infections, potentially overcoming antibiotic resistance. This combined therapy approach represents a valuable strategy for treating drug-resistant bacteria and ensuring optimal patient outcomes [87].

While the potential of EOs in medical device coatings is promising, further research is still needed to fully understand their mechanisms of action and optimize their effectiveness. Factors such as the choice of EOs, concentration, and method of application all play a crucial role in determining the success of these coatings. Additionally, the long-term safety and stability of essential oil coatings need to be thoroughly evaluated to ensure the well-being of patients. Thus, while EOs show promise in preventing hospital-acquired infections by reducing bacterial buildup on medical devices, more research is needed to ensure their safe and effective use in clinical settings.

Hammer et al. [88] conducted a comprehensive examination to determine the extent to which *Melaleuca* oil, also known as tea tree oil, and its major antimicrobial component, terpinen-4-ol, prevent the adherence of *S. aureus* to various surfaces. In this study, slides were meticulously coated with a solution of these oils and subsequently dried prior to being brought into contact with a suspension of the bacterial strain. To accurately quantify the bacterial adherence, the researchers employed a highly sensitive micro-balance, capable of measuring mass changes to the nearest 0.0001 g. Astonishingly, the application of *Melaleuca* oil demonstrated a remarkable ability to prevent the adherence of *S. aureus* to the slides in a concentration-dependent manner. Remarkably, when a concentration of 1 mg/cm^2^ of terpinen-4-ol was employed, the prevention of adherence was so potent that no measurable mass increment was observed on the slide itself. This finding signifies the immense potential of both *Melaleuca* oil and terpinen-4-ol in mitigating *Staphylococcus aureus*-associated infections related to biofilm formation on catheters and other medical devices. Moreover, it is noteworthy to mention that these oils exhibited no discernible disfigurement or alteration in the physical structure of the slides. Implications of this research are far-reaching and hold significant promise for the development of novel strategies to combat biofilm-associated infections caused by *S. aureus*, benefiting countless individuals worldwide.

Furthermore, the study conducted by Nova et al. [89] sheds light on the crucial need for effective prevention strategies against *S. aureus*-associated infections [89]. Biofilm formation on medical devices, such as catheters, poses a serious threat to patient health and can lead to life-threatening complications. The fact that *Melaleuca* oil and terpinen-4-ol exhibit such potent antimicrobial properties highlights their potential as powerful tools in combating these infections. By effectively preventing bacterial adherence, melaleuca oil and terpinen-4-ol offer a promising avenue for reducing the risk of biofilm-related infections and improving patient outcomes. In addition to their antimicrobial activity, *Melaleuca* oil and terpinen-4-ol also demonstrated excellent biocompatibility with the tested slides. This is a crucial consideration in the development of any antimicrobial intervention, as it is important to ensure that the treatment does not cause harm or damage to the host material. The fact that these oils did not result in any discernible disfigurement or alteration in the physical structure of the slides suggests that they could be seamlessly integrated into existing medical devices without compromising their functionality or safety [89]. To conclude, the study underscores the potential of *Melaleuca* oil and its major antimicrobial component, terpinen-4-ol, in preventing the adherence of *S. aureus* to various surfaces [90]. Their concentration-dependent efficacy, lack of adverse effects on the host material, and broader implications in the field of infection control make them valuable candidates for the development of novel strategies to combat biofilm-associated infections caused by *S. aureus*.

### 7.1. Challenges in Developing Effective Coatings

The formation of biofilms and bacterial resistance to antibiotics are the most critical and challenging issues in the clinical use of medical devices, leading to various complications and compromised patient care. As a result, there is an urgent need to address these problems by implementing innovative strategies and developing novel approaches. In order to ensure the future enhancement of patient care, it is imperative to focus on the development of new antimicrobial agents that are not only highly potent but also safe for clinical use. These agents should exhibit exceptional efficacy in eradicating bacterial colonization and preventing the formation of biofilms on medical devices.

Furthermore, it is essential to thoroughly evaluate the potential of existing antimicrobial agents in combating bacterial colonization and biofilm formation. By conducting comprehensive studies and carefully analyzing their effectiveness, the agents that show promise in preventing device-associated infections can be identified. The evaluation process should encompass a wide range of medical devices, including but not limited to catheters, implants, prosthetics, and other healthcare equipment. The aim is to provide clinicians and healthcare professionals with a comprehensive understanding of the most effective methods to prevent device-related infections and ensure patient safety. Moreover, investing in research that delves deep into the mechanisms of bacterial resistance to antibiotics is crucial. By unraveling the complex pathways and genetic factors involved in this resistance, one can develop innovative therapies that can effectively target and overcome these challenges. By dedicating ample resources, funding, and research efforts to these crucial areas, it is possible to create a healthcare landscape that is characterized by enhanced device safety, reduced infections, and ultimately, improved patient outcomes [68,69,70,91,92,93].

Impregnating or coating medical devices with antibiotics has been largely ineffective due to issues with efficacy, biofilm penetration, prolonged antibiotic release, and the emergence of resistant organisms. These challenges have led to significant interest in developing alternative non-antibiotic methods for preventing device-related infections. Researchers are exploring antimicrobial peptides, novel biomaterials, innovative surface modifications, and advancements in nanotechnology to create environments hostile to bacterial attachment and biofilm formation. Additionally, strategies that harness the body’s immune system are being investigated. These multidisciplinary approaches aim to revolutionize infection prevention and improve patient outcomes [94].

### 7.2. EO Formulations for Coatings

In the next section, the specific formulations of EOs for coatings, detailing thin film formation, properties, and benefits in antimicrobial protection, are presented.

Using EOs as a natural method to combat bacterial infection is starting to gain significant interest among researchers due to their remarkable effectiveness. Numerous studies have provided substantial evidence of the efficacy of EOs in treating infections, showcasing their potential to revolutionize the field of infection control. However, their potential to provide infection-resistance properties to biomaterials and medical devices remains largely unexplored, presenting a fascinating avenue for further investigation and immense potential for enhancing the efficacy of medical devices and biomaterials in combating bacterial infections. Minimal research has been conducted on EO elution from various carriers, which limits our understanding of their sustained release capabilities and hinders their widespread implementation [95]. Currently, there is no well-defined EO sustained release system available, highlighting a gap in the field. Developing a systematic approach to achieve sustained antibacterial activity from EOs is crucial to fully harness their potential as natural remedies. For EOs to exert prolonged antibacterial effects, they must be released at specific concentrations. Controlling the release of EOs is essential to maximize their antibacterial efficacy while ensuring safety. Maintaining optimal concentration levels is critical; insufficient concentrations may render the oil ineffective against bacteria, whereas excessive concentrations could harm surrounding tissues. Further research is needed to explore the elution process and identify suitable carriers for EOs, advancing their application in medical treatments [96].

Currently, the most commonly employed carrier for the sustained release of EOs is hydroxypropylmethyl cellulose. The methodology of the preparation of the oil–cellulose system directly combines the oils with the cellulose. This results in a product for which there is no gradient in the concentration of oil eluted; the initial concentration is too high and may be toxic, becoming ineffective when the cellulose degrades and releases the remainder of the oil. An attempt to elute a stable and consistent concentration of oil involves microencapsulation. This has been described using ultrasound in the preparation of oil-in-water emulsions to produce alginate-chitosan microcapsules. Studies on the elution characteristics have proved favorable, displaying a ten-day release of thyme oil with cinnamaldehyde against *P. fluorescens*. This could be a potential system to elute oils at concentrations effective in eradicating bacteria without having cytotoxic effects. The microcapsules could be added to a film-forming polymer of the EOs to create a thin antibiotic coating [97].

### 7.3. Various Approaches for Integrating Essential Oils into Coatings

In this section, various approaches for integrating EOs into coatings, a method increasingly being explored for its potential to add antimicrobial, fragrant, or therapeutic properties to surfaces, are outlined. Table 4 considers different techniques and their implications.

Each methodology offers distinct advantages based on the application’s specific needs, ranging from enhancing product longevity to imbuing surfaces with health-related features. These integration strategies highlight the versatility of essential oils beyond their traditional uses and underscore the potential for innovative applications in various industries.

Encapsulation in a polymer coating is an effective and widely used technique in various industries. It serves the vital purpose of enclosing EOs within a cavity in a polymer or microcapsule, enabling the controlled release of the oils. This technique has proven to be optimal in terms of performance, as long as the coating is defect-free, chemically resistant, and capable of achieving the desired release rate of the payload. The use of microencapsulation has become increasingly prevalent in different industries, and there are currently numerous methods available for producing microcapsules containing essential oils, which can then be used as highly effective antimicrobial coatings. Over the course of time, the production of microcapsules has evolved and expanded significantly, resulting in a wide range of available methods that cater to diverse needs [98].

Some of these methods include spray-drying, fluidized bed coating, in situ polymerization, simple and complex coacervation, and phase separation. Notable studies conducted by Li et al. (2021) [105], Li et al. (2023) [106], Özbek et al. (2004) [107], and many other respected researchers have explored and experimented with various encapsulation methods, showing the immense potential and versatility of these techniques. In a noteworthy study conducted by Raymond et al. (2020) [108], lemongrass oil was encapsulated into pectin using the emulsion and coacervation method. The resulting pectin film was subsequently subjected to antimicrobial activity testing against black gram seed. The study effectively demonstrated the effectiveness of this encapsulation method in preserving the antimicrobial properties of the essential oil. Similarly, Rajkowska et al. (2014) [109] employed the cutting-edge nanoencapsulation technique to effectively preserve the antibacterial properties of tea tree oil and thyme oil. They utilized polycaprolactone (PCL) and polyurethane (PU) to create nano-thin films, which were then tested against *E. coli*. The results showed significant activity against the bacteria, further validating the potential of encapsulation in antimicrobial coatings. To conclude, the utilization of encapsulation in polymer coatings for EOs offers unprecedented potential in the development of antimicrobial coatings [110].

## 8. Evaluation of EO Coatings

Recent research has highlighted the potential value of EO coatings in developing antimicrobial surfaces. Another benefit of these coatings would be the combination of EOs with repellent or antimicrobial agents, which would act synergistically to provide a composite layer with multiple functions in repelling insects, bacteria, fungi, or algae. This would be particularly useful for nets, tents, outdoor medical equipment, etc. Currently, these findings are in their early stages, so the testing is minimal, and to date, no field trials have been commissioned under real-use conditions. However, based on preliminary research, it is evident that EO coatings have immense potential to revolutionize the field of antimicrobial technology by offering a natural and environmentally friendly alternative to traditional synthetic coatings. By harnessing the power of nature, one can develop surfaces that not only prevent the growth and spread of harmful microorganisms but also provide additional benefits such as insect repellency and resistance to algae formation. The versatility of EOs opens possibilities in various industries, ranging from healthcare to agriculture. However, it is important to note that further research and extensive field trials are necessary to fully understand the long-term effectiveness and durability of EO coatings. Only through rigorous testing can EOs meet the highest standards of safety and performance [110,111].

**Scheme 1:**
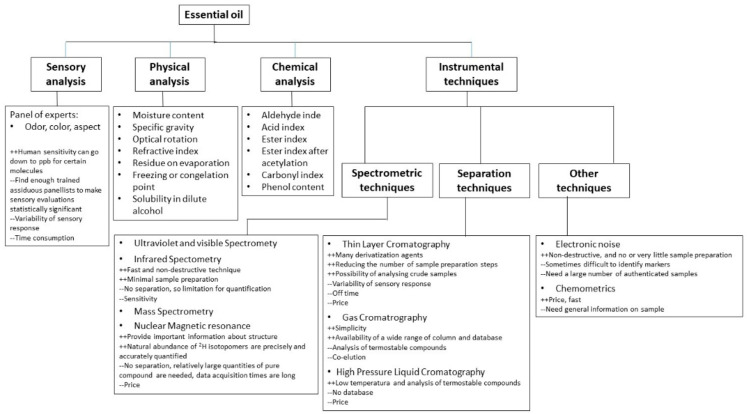
Summary of the different procedures used for evaluating the quality of essential oils, together with some of their main advantages and disadvantages [7].

In this regard, the potential of EO coatings as powerful antimicrobial agents to combat these resistant strains and impede the formation of biofilms has been thoroughly examined (Scheme 1).

Moreover, it is worth noting that chemical methods incorporating leaching additives have demonstrated limited success in this battle against antibiotic resistance [111]. In contrast, it is widely believed that a long-term treatment involving the surface application of oils can yield superior results. By utilizing oils as a protective coating, one can establish a formidable defense against the proliferation of antibiotic-resistant strains and the formation of harmful biofilms.

(i) Testing antimicrobial efficacy

The antimicrobial efficacy of EO-containing coatings against methicillin-resistant *S. aureus* (MRSA) has been extensively investigated using a combination of established testing methods, including the European standard quantitative suspension test (EN1040) [112] and the Japanese Industrial Standard (JIS Z 2801) [113]. These tests evaluate the coatings’ antimicrobial activity by comparing viable microbial colonies from treated and control samples. The minimum inhibitory concentration (MIC) method, which identifies the lowest concentration needed to inhibit MRSA growth, is also crucial for assessing the practical effectiveness of these coatings. Lower MIC values indicate higher antimicrobial activity, demonstrating the potential of EO-containing coatings in combating resilient bacteria such as MRSA [114,115,116].

(ii) Assessing biocompatibility and cytotoxicity

An important step in the development of a medical device or coating is the assessment of its biological safety. With EOs possessing a wide range of activities, particularly antibacterial properties, toxicity can be an issue. This is especially pertinent to the issue of the development of an ALE (Active Layered Extract) coating at the concentration necessary to elicit antibacterial effects; it is important to check whether the coating is directly toxic to certain cell types or whether it elicits toxicity to cells in the long term in the form of apoptosis or necrosis. At the current time, there is an absence of an ISO standard for testing natural substances.

The MTT assay is a standard method for assessing cell viability but has limitations. Using flow cytometry can provide more detailed information on cell death mechanisms and the impact of the ALE coating on cellular processes. This advanced approach offers a deeper understanding of the biological effects of the coating, helping researchers to explore its impact on cell viability at a detailed level. Combining flow cytometry and microarray analysis provides a comprehensive approach to understanding the biological effects of cytotoxic oil mixtures in ALE coatings. This methodology allows researchers to characterize gene expression patterns and assess cell viability, cell death mechanisms, and cell cycle disruptions. This integrated analysis enhances the development and safety of medical devices and coatings by offering deep insights into the ALE coating’s bioactivity and its therapeutic potential while ensuring the safety of surrounding tissue [117].

(iii) Long-term stability and durability studies

An ideal coating must consistently release antibacterial agents over time for sustained protection, reducing the need for frequent reapplications and enhancing patient compliance. It should also exhibit broad-spectrum activity to target various pathogenic microbes, reducing the emergence of drug-resistant strains. Furthermore, these coatings must be selectively toxic to harmful microbes while preserving the integrity and function of mammalian cells, ensuring overall well-being and minimizing potential harm. This balance maximizes the benefits of antibacterial coatings in healthcare settings [118]. The successful implementation of highly effective antibacterial coatings could revolutionize antibiotic therapy and infection control practices. This technology offers the potential to reduce or even eliminate the need for antibiotics, enhancing healthcare outcomes and significantly curtailing the risk of developing antibiotic resistance. By diminishing reliance on conventional antibiotics, these coatings can prevent the emergence of untreatable infections caused by antimicrobial resistance. With their ability to release a steady and effective concentration of potent antibacterial agents, exhibit an extensive and inclusive broad spectrum of activity against diverse microbial strains, and preserve the well-being and viability of mammalian cells, these coatings have the exceptional potential to substantially mitigate the impact and consequences of infections caused by drug-resistant bacteria and other microbial agents [119,120].

The mode of action of antibacterial coatings based on EOs developed by Chivandi [121] is still under investigation, but it is known that the antibacterial activity of the coatings was only observed when EOs were added directly to the paint formulation and not when they were coated with a solution of essential oils. An acceptable release profile has been observed for several EOs with successful antimicrobial activity, with volatile components released over a period of weeks and other constituents with longer-lasting antibacterial activity being released over months. The release of oil from the coatings is dependent on water diffusing into the coating and is possibly due to oil segregating to the surface to reform an oil phase, from which bacterial toxicity is maintained as the oil continues to leach out. This is an advantage over antibacterial coatings that release the antibacterial agent too quickly and this ability to control the rate of release can be manipulated by altering the type of oil and oil content in the coating. An example of this was observed in a study where the antibacterial activity of coatings containing pine oil was negatively affected by increasing turpentine content [121].

## 9. Applications of Essential Oil Coatings

EOs have shown effectiveness in treating infected wounds and preventing biofilm formation on various surfaces, including consumer products and medical supplies such as wound dressings and catheters [122].

Next, the literature review will explore the various applications of essential oil coatings, highlighting their effectiveness and versatility in different environments.

## 10. Applications of EO-Based Coatings

### 10.1. Medical Devices and Implants

The problem of microbial adhesion and subsequent colonization of medical devices and implants has been widely recognized in the medical field. The consequences of such colonization can be severe, often leading to the development of infections that pose a significant risk to patients. Consequently, there is an urgent need to devise effective strategies to reduce microbial adhesion on these devices and prevent the associated complications. In light of this pressing issue, our study focused extensively on the utilization of natural plant compounds as potential solutions.

Among the numerous compounds investigated, thyme and cinnamon EOs emerged as highly promising candidates due to their exceptional properties. These natural oils possess unique antibacterial characteristics that make them ideal agents for preventing the colonization of medical devices by pathogenic bacteria, which is a critical step in the development of infections. By harnessing the inherent power of these natural oils, the aim is to create a formidable barrier that effectively inhibits bacterial adhesion and subsequent growth on the surfaces of medical devices. Through rigorous experimentation and thorough analysis, the findings conclusively demonstrated the efficiency of thyme and cinnamon essential oils in inhibiting the colonization of medical devices by pathogenic bacteria. The underlying mechanisms behind their effectiveness involve disrupting the biofilm formation process, a key step in bacterial adhesion and growth. Additionally, these oils exhibited potent antimicrobial activity, further hindering the colonization process and diminishing the overall risk of infections associated with the use of medical devices [123].

Using a parallel plate flow chamber, researchers investigated the adhesion behavior of *S. epidermidis* on polymer surfaces in the presence of a continuously flowing nutrient medium. This microorganism is a known cause of infections related to medical devices. The study revealed that *S. epidermidis* swiftly adhered to the polymer surface and colonized it within two days. To counter this, the chamber was treated with a solution of EOs dissolved in 70% alcohol, which significantly inhibited the colonization of *S. epidermidis*. Further experiments incorporating essential oils into a hydrogel system also showed substantial inhibitory activity against various strains of *S. epidermidis* using agar diffusion tests, highlighting the potential of essential oils in reducing the risk of infections from medical device use [124].

In the context of examining microbial adhesion and colonization on medical devices, studies have demonstrated that immature cultures of microorganisms, particularly *S.epidermidis*, can form dense biofilms on surfaces using modified Robbins devices [109]. This biofilm formation is a critical factor in developing infection models for medical research. EOs have shown potential in preventing initial biofilm formation on polymer surfaces, suggesting that sustained-release biofilm models could be valuable for testing antimicrobial agents’ effectiveness in preventing infections linked to medical devices [125].

A study was conducted by Puiu RA et al. [126] to compare the selective cytotoxicity of silver-based nanocoatings on MCF7 tumor cells and normal VERO cells. The following nanocoatings were created using the matrix-assisted pulsed laser evaporation (MAPLE) method and were infused with five cytostatic drugs (doxorubicin, fludarabine, paclitaxel, gemcitabine, and carboplatin) and five EOs (oregano, rosemary, ginger, basil, and thyme). Various techniques such as X-ray diffraction, thermogravimetry, Fourier-transform IR spectroscopy (FTIR), IR mapping, and scanning electron microscopy (SEM) were used to analyze these nanocoatings. Their impact on the MCF7 tumor and normal VERO cell lines was studied using cell viability MTT and cytotoxicity LDH assays. The study discovered that all nanocoatings containing antitumor drugs displayed potent cytotoxic effects on both tumor and normal cells. However, nanocoatings embedded with silver nanoparticles (AgNPs) and loaded with rosemary and thyme essential oils exhibited remarkable and statistically significant selective cytotoxicity against the tested cancer cells. Furthermore, the essential oil-loaded nanocoatings were assessed for their antimicrobial and antibiofilm activity against *S. aureus*, *E. coli*, and *C. albicans*. The nanocoatings containing ginger and thyme essential oils demonstrated the most effective antibiofilm activity, while all EO-based nanocoatings significantly reduced cell viability for all studied pathogens.

Bone has a remarkable ability to heal and remodel itself. However, bone diseases are increasingly common in the elderly population, posing a significant burden on individuals and society. Traditional treatments such as surgical procedures, bone grafts, and transplants can disrupt bone regeneration processes and lead to infections. To address this, researchers are exploring bone-regenerative strategies and identifying therapeutic agents to aid the bone-healing process. Some studies are looking into the direct application of essential oils on bone tissue or using them as bioactive compounds in bone scaffolds or coatings for bone implants. EOs under investigation include St. John’s wort, rosemary, thyme, ylang ylang, white poplar, eucalyptus, lavender, and grape seed. This paper [127] aims to provide an overview of the main mechanisms involved in bone repair and regeneration and the potential of essential oils to enhance these mechanisms.

The study by Gherasim et al. [128] focuses on the development and evaluation of composite coatings made of polylactic acid (PLA) embedded with iron oxide nanoparticles (Fe_3_O_4_) modified with Eucalyptus globulus essential oil. Magnetite particles conjugated with a natural Eucalyptus antibiotic (Fe_3_O_4_@EG) were synthesized using the co-precipitation method. The composition and microstructure of the particles were investigated using various techniques such as grazing incidence X-ray diffraction (GIXRD), FTIR, thermogravimetric analysis (TGA), transmission electron microscopy (TEM), and dynamic light scattering (DLS). The PLA/Fe_3_O_4_@EG thin films were obtained using the MAPLE technique. The experimental conditions for laser processing were optimized using complementary infrared microscopy (IRM) and SEM investigations. The biocompatibility of the composite coatings with eukaryote cells was evaluated using mesenchymal stem cells. Additionally, the anti-biofilm efficiency of composite PLA/Fe_3_O_4_@EG coatings was tested against Gram-negative and Gram-positive pathogens in vitro. The study discusses the successful synthesis of PLA/Fe_3_O_4_@EG nanocomposite coatings and their potential use as biocompatible and anti-biofilm surfaces for implantable biomaterials and devices. Using a modified co-precipitation protocol, ultra-small (7.5 ± 2.5 nm) highly crystalline magnetite nanoparticles were combined with a natural antibiotic, Eucalyptus essential oil. The MAPLE technique was used to efficiently, uniformly, and stoichiometrically synthesize nanostructured composite thin films. Cellular assays demonstrated that the PLA/Fe_3_O_4_@EG coatings did not affect the viability and proliferation of eukaryote cells, while significantly interfering with the formation and maturation of bacterial biofilms. Therefore, the proposed PLA/Fe_3_O_4_@EG nanocomposite coatings are suitable for modifying the surfaces of implantable biomaterials and devices to enhance their biocompatibility and induce or enhance their anti-infective effects.

In another study by Anghel et al. [129], Fe_3_O_4_ nanoparticles of 9.4 nm in size, functionalized with *Cinnamomum verum*, were transferred onto gastrostomy tubes (G-tubes) using the matrix-assisted pulsed laser evaporation (MAPLE) technique. The purpose of this was to evaluate their antibacterial activity against both Gram-positive and Gram-negative microbial colonization. X-ray diffraction analysis of the nanoparticle powder showed a polycrystalline magnetite structure, while infrared mapping confirmed the integrity of C. verum functional groups after the laser transfer. The deposited films had a specific topography, consisting of a uniform thin coating along with several aggregates of bio-functionalized magnetite particles that covered the G-tubes. Cytotoxicity assays showed that the G-tube surface biocompatibility increased after Fe_3_O_4_@CV treatment, allowing for normal endothelial cell development for up to five days of incubation. Microbiological assays on nanoparticle-modified G-tube surfaces demonstrated improved anti-adherent properties, significantly reducing colonization by both Gram-negative and Gram-positive bacteria.

The aim of the study by Grumezescu et al. [130] was to demonstrate the effectiveness of using Fe_3_O_4_ core/shell nanostructures to stabilize an *Eugenia carryophyllata* EO on catheter surface pellicles in order to improve their resistance to fungal colonization. Microwave-assisted extraction was used to extract the EO from *Eugenia carryophyllata* using a Neo-Clevenger device, and its chemical composition was determined through GC-MS analysis. Fe_3_O_4_/oleic acid-core/shell nanoparticles were synthesized through a precipitation method under microwave conditions. The NPs were characterized using high-resolution TEM and further processed to achieve a core/shell/EO coated-shell nanosystem which was used to coat the inner surface of central venous catheter samples. The tested fungal strains were recently isolated from different clinical specimens. Confocal laser scanning microscopy (CLSM) was used to assess the biofilm architecture. The results suggest that the hybrid nanomaterial (core/shell/coated-shell) can effectively stabilize *E. carryophyllata* EO, which prevented or inhibited fungal biofilm development on the functionalized catheter. This highlights the potential of using these nanosystems to obtain improved anti-biofilm coatings for biomedical applications.

Fungal biofilms can resist many antimicrobial agents, making it difficult to treat fungal infections. Researchers conducted a study [131] to create a nanobiosystem using nanoparticles and Rosmarinus officinalis essential oil to prevent *C. albicans* and *C. tropicalis* from forming biofilms on catheter surfaces. The EO was extracted from *R. officinalis* and its composition was determined using GC-MS analysis. Oleic acid-coated Fe_3_O_4_ nanoparticles were synthesized using microwave conditions and applied to the catheter surface using a magnetic field. The EO was then applied by adsorption. The researchers tested both the coated and uncoated catheters using culture-based methods and confocal laser scanning microscopy. The results showed that the nanobiosystem strongly inhibited the ability of *Candida* strains to adhere to the catheter surface, which could be useful in preventing infections related to prosthetic devices.

### 10.2. Wound Dressings and Bandages

An ideal wound dressing would encompass a variety of exceptional qualities to ensure optimal healing and protection. Firstly, it should maintain a moist environment that prevents the dissemination of bacterial pathogens, safeguarding against potential infections. In addition, this dressing should possess non-adherent, non-toxic, and non-allergic properties to promote compatibility with the patient’s skin and minimize the risk of adverse reactions. Furthermore, it should exhibit outstanding thermal insulation capabilities, effectively preserving the wound’s temperature for enhanced recovery. Moreover, the dressing should facilitate efficient hemostasis, swiftly stopping any bleeding that may occur. Simultaneously, it should allow for unhindered movement, ensuring convenience and comfort for the patient during their healing process. A crucial aspect to consider is that this dressing should be entirely free from any particulate matter and fibers. This meticulous cleanliness and hygiene will prevent any further complications or damage to the wound, granting the highest level of care possible. Furthermore, this remarkable dressing should also possess effortless removal properties, eliminating any potential discomfort or pain for the patient. It should be gentle enough to be peeled off smoothly, without causing any trauma or tissue damage. Regrettably, despite numerous advancements in the field of wound dressings, no existing products have been able to encompass all of these highly advantageous properties and characteristics. This realization highlights the need for further research and innovation to develop a dressing that truly meets all these essential requirements, ushering in a new era of wound care excellence [132].

There is a clear and distinct division between modern wound dressings and their traditional counterparts. Traditional dressings, which have been in use for centuries, typically involve the utilization of basic materials such as gauze, lint, plasters, and creams to effectively cover and protect wounds. In comparison, modern techniques have brought innovative options in the form of film, foam, and hydrocolloid dressings. These cutting-edge dressings differ greatly from their traditional counterparts, as they are crafted using advanced manufacturing processes and sophisticated materials. It is worth noting that the vast majority of traditional wound dressings are closely linked to natural sources, with some even directly utilizing plants as their primary material (e.g., lint) [133]. However, it is important to recognize that despite their long usage, many traditional dressings possess limited antimicrobial activity. One significant drawback associated with the use of gauze and lint is that they often leave behind residual materials within the wound when attempting to change dressings. This can subsequently elevate the risk of infection as the remaining fibers provide an ideal environment for microbial colonization and infection. On the other hand, modern film and hydrocolloid dressings have departed from reliance on natural sources and instead incorporate synthetic materials. Unfortunately, this means that they often overlook the potential benefits and antimicrobial properties offered by EOs. Nonetheless, recent advancements in microencapsulation technology have opened up new possibilities. It is now conceivable that EOs can be encapsulated within a polymer-coated film and released gradually, thus blending the benefits of modern dressings with the antimicrobial potency of EOs. The development of essential oil-loaded modern dressings holds particular promise in facilitating the healing of chronic wounds and leg ulcers that exhibit excessive exudation and necrotic tissue. By harnessing the therapeutic potential of essential oils, these dressings can aid in the profound healing of challenging wounds that have proven to be resistant to conventional treatments. Moreover, it is essential to emphasize that any dressing designed to possess antimicrobial capabilities must still fulfill the other fundamental attributes of an ideal dressing. These include factors such as promoting moisture balance, protecting the wound from external contaminants, facilitating gas exchange, maintaining a proper temperature, minimizing pain and discomfort, and ensuring ease of application and removal. Only when all these qualities are present can a dressing be regarded as truly optimal in its purpose [134].

### 10.3. Food Packaging and Preservation

Edible coatings have been extensively utilized in the food industry for various purposes and with different materials. These materials include proteins, polysaccharides, and lipids, all of which have been employed to enhance the shelf life and overall quality of food products. Among the techniques used in this regard, antimicrobial coatings have gained significant attention as they provide a food-compatible approach to reduce surface and internalized microbial loads in processed foods. Numerous studies have investigated the incorporation of EOs into edible films and coatings to inhibit the growth of foodborne pathogens. For instance, early research focused on the antifungal properties of cinnamon oil when incorporated into a chitosan coating, resulting in the successful inhibition of *Penicillium expansum* on apples [135]. Another example involves the utilization of antimicrobial basil EOs in alginate coatings, effectively preventing the growth of Aeromonas hydrophila on fish stored at 5 °C. Furthermore, recent findings have demonstrated that chitosan coatings containing cinnamon and allspice essential oils exhibit remarkable inhibition capabilities against *Listeria monocytogenes* [136]. Biofilm formation occurs when bacteria adhere to a surface and secrete an extracellular matrix, typically slimy and composed of polysaccharides. This matrix serves as protection for bacteria against adverse environmental conditions and acts as a barrier to prevent the penetration of antimicrobial agents. Importantly, biofilm cells have been found to be significantly more resilient to antibiotics than their planktonic counterparts, with resistance levels ranging from 10 to 1000 times higher. This poses a significant challenge in the food industry, as foodborne pathogens can form biofilms on processing equipment and storage surfaces, leading to persistent contamination of food products. Additionally, reports have indicated the presence of antibiotic-resistant strains, such as MRSA and VRE isolates, in biofilm samples obtained from poultry processing equipment. To mitigate the problem of biofilm formation and the associated antibiotic resistance, the use of antimicrobial coatings presents a promising solution. These coatings can effectively prevent the formation of biofilms on food products by significantly reducing the microbial load. Moreover, the increased susceptibility of biofilm cells to EOs means that antibiotics are ineffective for treating biofilm-contaminated food products. Coatings containing essential oils offer a wide range of antimicrobial properties and are particularly suitable for organic food products, as they are derived from natural and renewable resources. By utilizing these innovative coatings, the food industry can ensure the safety and quality of their products while also preserving the overall integrity of the environment and promoting sustainable practices [137].

## 11. Future Directions and Challenges

Coating with both EOs and antibiotics to evaluate synergy is a potential next step to further understanding the relationships between them. It would be intriguing and highly informative to investigate whether the incorporation of a sublethal dose of antibiotics, in conjunction with the powerful EOs, could tremendously enhance the efficacy of the current treatment, thus potentially impeding the relentless emergence of antibiotic resistance. This novel approach necessitates meticulous investigation on a considerably advanced model, surpassing the limitations of a mere petri dish. It is reasonable to ponder that the gradual elution of EOs from the exquisite coating could potentially exert a notable influence on the antibiotics, whose release from the biomaterial is comparably slower. Therefore, further exploration is warranted to comprehensively elucidate this intricate interplay. One striking possibility that emerges is the assembly of a dynamic complex comprising the EOs and antibiotics, ideally resembling the structure of an antibiotic with an inherently more lipophilic moiety. Such a captivating innovation could serve as the bedrock for the development of a cutting-edge modified antibiotic-eluting coating, ingeniously designed to triumph over the hurdles posed by the upregulation of multidrug efflux pumps, which contribute significantly to the antibiotic resistance phenomenon. Undoubtedly, this combination could be a breakthrough in the battle against antibiotic resistance, paving the way for more effective therapeutic strategies and ultimately benefiting the health and well-being of countless individuals worldwide [138].

The research will focus on optimizing the incorporation of EOs into coatings to enhance their effectiveness and exploring modifications to increase their antibacterial potency. For instance, nanoparticles such as nano-silver have demonstrated heightened antimicrobial activity compared to conventional silver, even at lower concentrations. An EO can be incorporated into a silver nanoparticle through the same method that has already been achieved to create an altered release of drugs to prevent restenosis with anticancer drugs in eluting stents. Testing with drugs can follow a similar method to antibiotic-eluting coatings today and would overcome the problem of drug-eluting catheters, for example, where pure heparin bonding does not last for the 7 days required for a catheter to be effective. An antimicrobial therapeutic agent can still be the better treatment option for certain diseases or conditions [139].

### 11.1. Potential for Combination Therapies

A major advantage of an EO delivery system is the potential to use it in combination with other antimicrobial agents, for instance, antibiotic drugs. EOs have the potential to act in ways that are synergistic with antibiotics, thereby reducing effective antibiotic concentrations and combating resistance, and additive, thus broadening the antimicrobial spectrum. Studies have already shown synergy between a variety of oils and antibiotics against different strains of bacteria. The synergy is likely due to oils affecting bacterial resistance factors in such a way as to allow antibiotics to function effectively. For instance, the oil of *Melaleuca* has been shown to inhibit cell surface hydrophobicity and, in turn, increase outer membrane permeability, thereby potentiating the activity of various antibiotics [140].

Synergistic and additive combinations could possibly be identified by in vitro bioassays designed to measure the effects of oils and antibiotics in a systematic manner, then further explored by animal model studies. By determining specific combinations of oils, antibiotics, and dosing regimens, it may be possible to apply these therapies to clinical medicine. A particularly exciting possibility is the use of EOs to reverse resistance to antibiotics. For instance, the oil of *Pogostemon cablin* has been shown to inhibit ESBL-producing *E. coli* isolated from clinical specimens. The ability of oils to break down resistance mechanisms in bacteria is an area that requires further research. If resistance to oils is minimal and oil mechanisms are different from specific antibiotics, it may be possible to use oils in combination to restore antibiotic susceptibility to resistant strains. This would be a valuable outcome considering the lack of efficacy of certain antibiotic treatments and the shortage of antibiotic development [141].

In other studies, Valente et al. reported synergistic antifungal activity between *Mentha piperita* and *Mentha spicata* EOs when combined with conventional antifungal agents against *Candida* spp. [142]. Additionally, Langeveld et al. (2014) [138] highlighted the synergistic interactions between EOs and antibiotics, such as carvacrol with ampicillin and thymol with chloramphenicol, resulting in enhanced antimicrobial effects against multidrug-resistant bacteria. Furthermore, Carson et al. demonstrated the synergistic bactericidal activity of *Melaleuca alternifolia* (tea tree) oil with various antibiotics against *Staphylococcus aureus*, suggesting the potential of EOs as an adjunctive therapy to combat bacterial infections [143].

Research has demonstrated the synergistic antibacterial activity of *Cinnamomum verum* (Ceylon cinnamon) EO when combined with sertraline, a selective serotonin reuptake inhibitor. This combination exhibited significant growth inhibition across a range of Gram-positive and Gram-negative bacteria, showcasing a powerful synergy that could mitigate the spread of drug-resistant pathogens [144]. A study investigated the antibacterial and antibiofilm activities of linalool, carvacrol, and eugenol essential oils against MRSA and vancomycin-resistant Staphylococcus aureus (VRSA). These essential oils demonstrated significant synergistic interactions with traditional antibiotics such as cefoxitin and vancomycin, effectively converting resistant strains to sensitive ones [145]. Another study evaluated the synergistic effects of orange and petitgrain EOs with antibiotics against *Escherichia coli* and MRSA. The modified well diffusion method and checkerboard assay revealed high-level enhancement combinations, underscoring the potential of these essential oils to boost antibiotic efficacy significantly [146].

The combination of *Thymus capitatus* (thyme) and *Syzygium aromaticum* (clove) EOs with antibiotics was shown to exhibit potent synergistic effects against multi-resistant Salmonella strains. This synergy reduced the minimum effective dosage of both essential oils and antibiotics, highlighting their potential in treating stubborn infections [147].

The synergistic antimicrobial interaction of peppermint and lavender EOs was studied, showing that their combination with antibiotics could broaden the spectrum of activity and reduce the required doses of antibiotics. This combination was particularly effective against various bacterial pathogens, offering a promising alternative for enhancing antibiotic therapy [145].

These examples highlight the potential of EOs to enhance the efficacy of antibiotics, providing a multifaceted approach to combat antimicrobial resistance. By exploring these synergistic combinations, researchers aim to develop more effective treatments for resistant infections, leveraging the natural antimicrobial properties of EOs.

### 11.2. Limitations and Potential Drawbacks

EOs have garnered significant attention for their antimicrobial properties, especially as potential alternatives in combating antibiotic resistance. However, despite their promising benefits, EOs come with certain limitations and potential drawbacks that need to be considered.

One significant limitation of EOs is their variability in composition. The chemical makeup of EOs can vary significantly depending on the plant species, geographical location, extraction method used, and storage conditions [11]. This variability can affect the consistency and predictability of their antimicrobial efficacy. For instance, the concentration of active components such as terpenes and phenylpropanoids can differ, leading to inconsistent results in their application.

Another limitation is the potential for cytotoxicity. While EOs exhibit strong antimicrobial properties, their high potency can also lead to toxicity in human cells. Studies have shown that certain EOs can cause skin irritation and allergic reactions. Additionally, while EOs are generally considered safe when used appropriately, there are concerns regarding their potential toxicity, particularly at high concentrations or when ingested [12]. Therefore, careful formulation and testing are required to ensure their safety and efficacy.

The stability of EOs is also a concern. EOs are volatile compounds, which means they can evaporate quickly and lose their effectiveness over time. This poses a challenge in creating long-lasting antimicrobial coatings and formulations [148].

To address this, researchers have explored encapsulation techniques and polymer coatings to improve the stability and sustained release of EOs. However, these methods add complexity and cost to the production process [11].

EOs also face regulatory and standardization challenges. The lack of standardized protocols for the extraction, formulation, and testing of EOs makes it difficult to ensure consistent quality and efficacy across different products and studies. Regulatory bodies need to establish clear guidelines to address these issues and facilitate the wider adoption of EOs in healthcare and other industries [149].

Furthermore, the development of resistance to EOs, although less common compared to synthetic antibiotics, remains a potential risk. Continuous and widespread use of EOs could potentially lead to the emergence of resistant strains of microorganisms, reducing their long-term effectiveness.

## 12. Conclusions

The use of EOs offers a promising approach to combat antibiotic resistance and meet the urgent need for effective antimicrobial treatments. This review highlights how EOs target bacterial microorganisms through unique mechanisms, distinct from traditional antibiotics. Despite challenges in fully understanding their mode of action, EOs have demonstrated significant efficacy against a wide range of bacteria. Exploring advanced coating solutions that incorporate EO formulations for medical devices underscores their potential as strong alternatives to synthetic antibacterial agents. This resurgence of interest in natural remedies emphasizes the importance of integrating traditional knowledge with modern scientific advances to address challenges in the post-antibiotic era. The comprehensive insights provided in this review offer valuable perspectives on the therapeutic potential of essential oils in enhancing antibiotic effectiveness and combating antibiotic resistance.

## Figures and Tables

**Table 1:** Advantages and disadvantages of essential oils extraction methods.

Extraction Method			Principle	Advantages	Disadvantages	Ref.
Conventional	Hydro distillation	Water distillation	The plant material is fully submerged in water that is then brought to a boil. It is crucial to continuously stir the plant material during the boiling process to prevent clumps of denser material from settling at the bottom of the container, where they can thermally degrade.	- Allows for the processing of finely powdered plant material or parts which, if exposed to live steam, would otherwise clump together, creating barriers that prevent steam penetration	- Complete extraction is not possible	[43]
		Water and steam distillation	The steam can be generated either in a satellite boiler or within the still, although separated from the plant material. The used equipment is similar to that used in the water distillation technique, but the plant material is placed above the boiling water on a perforated grid	- High oil production - More energy efficient than water distillation, as it is a faster process.	- Because of the low pressure of the steam, oils with a high boiling range necessitate a greater steam quantity, therefore requiring many distillation hours.	[8]
		Direct steam distillation	The plant material is distilled with steam generated outside the still, in a satellite steam generator (boiler).	- The amount of steam can be controlled - The plant material is not boiled above 100 °C, therefore avoiding thermal degradation - Large-scale oil production	This method requires more capital expenditure than the other two processes.	[44]
	Solvent extraction		Uses solvents such as hexane or ethanol to dissolve essential oils from plant materials. The solution is then evaporated to leave behind the oils.	- Efficient for extracting oils from delicate materials that cannot withstand heat	- Possible solvent residue in the oils, which may require further purification - Use of chemicals makes it less eco-friendly - Long extraction time and high solvent consumption	[45]
	Soxhlet extraction		- Uses a Soxhlet extractor where the solvent continuously cycles through the plant material to extract oils.	- Can extract oils completely - Good for small quantities of material - Efficient and thorough extraction	- Requires significant amounts of solvent -Time-consuming - Risk of thermal degradation of oils	[9]
	Cold pressing		Also known as expression or mechanical extraction, this method is primarily used for citrus essential oils such as orange, lemon, and lime. The rind of the fruit is mechanically pressed to release the oil.	- No heat is involved, which preserves the integrity of the oil. - Simpler and more natural process	- Limited to materials that easily release their oils (e.g., citrus peels) - Lower yields compared to methods that use heat or solvents	[46]
Innovative	Supercritical fluid extraction		Uses supercritical fluids, such as CO_2_, as solvents under high pressure and temperature to extract essential oils. The fluid penetrates the material and dissolves the volatile compounds, which are then collected after the pressure is reduced.	- No solvent residue - High purity and quality of oils - Efficient extraction of delicate compounds	- High initial cost of equipment - Requires precise control of temperature and pressure	[47]
	Microwave-assisted hydro distillation		Combines microwave heating with traditional hydro distillation. The microwave energy heats the water and plant material rapidly to release essential oils.	- Faster than traditional distillation - Reduced energy consumption	- Potential degradation of heat-sensitive compounds - Scale-up can be challenging	[48]
	Solvent-free microwave extraction		Utilizes microwave energy to directly heat the plant material without the use of water or any solvents, causing the cell walls to rupture and release essential oils.	- Low solvent use - Faster process than conventional distillation - Reduced thermal degradation of compounds	- Relatively new technique with limited data on large-scale application - Equipment and process control can be complex	[49]
	Ultrasound-assisted extraction		Uses ultrasonic waves to create micro-bubbles in a liquid medium. These bubbles collapse and create intense local pressure, disrupting cell walls and releasing essential oils.	- Low temperature process - Can be combined with other methods - Energy efficient	- May require longer extraction times - Scale-up requires careful optimization	[50]
	Microwave hydro diffusion and gravity		Applies microwave energy to vaporize water within the plant material. The vapor rises, capturing essential oils, and then condenses due to gravity in a collector.	- Low solvent use Faster process than conventional distillation - Reduced thermal degradation of compounds	- Relatively new technique with limited data on large-scale application - Equipment and process control can be complex	[51]

**Table 2:** Comparative analysis regarding the mechanism of action of EOs vs. conventional antibiotics.

	EOs	Antibiotics
Mechanism of action	Typically contain multiple bioactive compounds that disrupt bacterial cell membranes, inhibit enzymes, and interfere with pathogen metabolism. For example, thymol and carvacrol, found in thyme oil, are known to damage cell membranes and inhibit bacterial growth [54].	Usually target specific bacterial functions or structures, such as cell wall synthesis (e.g., beta-lactams), protein synthesis (e.g., tetracyclines), DNA replication (e.g., fluoroquinolones), or metabolic pathways (e.g., sulfonamides) [68].
Spectrum of activity	Often have broad-spectrum activity due to their multi-compound nature. For example, tea tree oil has been shown to be effective against a wide range of bacteria, including Gram-positive and Gram-negative strains [69].	Can be broad-spectrum (e.g., tetracyclines) or narrow-spectrum (e.g., penicillin). Broad-spectrum antibiotics can target a wide range of bacteria, while narrow-spectrum antibiotics are effective against specific types [70].
Resistance development	The complex mixture of compounds in EOs makes it more challenging for bacteria to develop resistance. Additionally, EOs can disrupt bacterial communication (quorum sensing), which is essential for resistance mechanisms [71].	Overuse and misuse of antibiotics have led to widespread antibiotic resistance. Bacteria can develop resistance through various mechanisms, such as producing enzymes that degrade the antibiotic (e.g., beta-lactamases) or altering the antibiotic’s target site [54].
Safety and side effects	Generally regarded as safe when used appropriately, but can cause allergic reactions and skin irritation in some individuals. High concentrations or improper use can lead to toxicity [71].	While effective, they can cause side effects such as gastrointestinal disturbances, allergic reactions, and, in some cases, more severe adverse effects such as organ toxicity. Long-term use can disrupt the natural microbiota, leading to secondary infections such as Clostridium difficile colitis [71].
Application and use	Used in various forms, such as topical applications, inhalation, or incorporated into products such as creams and lotions. Their versatility makes them suitable for both therapeutic and preventive measures [69].	Administered orally, intravenously, or topically, depending on the type and severity of infection. Their use is strictly regulated and requires medical supervision to prevent misuse and resistance development [54].

**Table 3:** Example cases of essential oil vs. synthetic antibiotics.

Studied Essential Oil vs. Antibiotic	Key Finding	Reference
Lavender oil vs. amoxicillin in treating bacterial infections	Lavender oil showed significant antibacterial activity, comparable to Amoxicillin in certain bacterial strains. This study evaluates the antimicrobial activity of lavender oil, particularly against *Staphylococcus aureus*, showcasing its potential as a complementary agent to traditional antibiotics.	[71]
Comparative study of cinnamon oil and ciprofloxacin	Cinnamon oil exhibited potent antibacterial effects, sometimes exceeding those of Ciprofloxacin, especially against *E. coli.* Focusing on the antimicrobial activity of cinnamon oil, this study highlights its efficacy against a spectrum of pathogens, including comparisons with traditional antibiotics.	[72]
Tea tree oil’s effectiveness vs. methicillin against MRSA infections	Tea tree oil was highly effective against MRSA strains, offering a viable alternative when conventional antibiotics such as Methicillin failed. This paper discusses the synergistic effects of tea tree oil when used alongside conventional antibiotics to combat MRSA infections, illustrating the oil’s potential in overcoming antibiotic resistance.	[73]
Eucalyptus oil versus vancomycin on *Staphylococcus aureus*	Eucalyptus oil showed potential as an antibiotic alternative, with strong antibacterial properties comparable to Vancomycin. Providing insight into the antibacterial properties of eucalyptus oil, particularly against MRSA and *E. coli*, this study supports the potential use of eucalyptus oil as an antibiotic alternative.	[74]
The role of peppermint oil vs. clarithromycin in treating *Helicobacter pylori* infections	Peppermint oil demonstrated a promising inhibitory effect on *H. pylori*, suggesting a potential role in treatment modalities. While focused broadly on the use of essential oils in aromatherapy, this review mentions the antimicrobial effects of peppermint oil, including potential applications against *H. pylori*.	[75]

**Table 4:** Various approaches for integrating essential oils into coatings.

Approach	Essential Oils Used	Application Surface	Integration Method	Key Benefits
Microencapsulation [98]	Lavender, Tea Tree	Textiles, Medical Devices	Encapsulation of essential oil in a polymer matrix	Controlled release, Enhanced durability, Protection of volatile compounds
Sol-Gel Process [99]	Thyme, Cinnamon	Glass, Ceramics	Dispersion of essential oils in a sol-gel to form a thin film	Antimicrobial properties, Chemical stability, Transparency
Layer-by-Layer (LbL) Assembly [100]	Peppermint, Eucalyptus	Fabrics, Packaging	Sequential deposition of polymers and essential oils	Customizable release profiles, Thin coating, Versatile application
Nanocomposite Coatings [101]	Citronella, Lemongrass	Outdoor Equipment, Walls	Incorporation into nanoscale fillers distributed in a polymer matrix	Improved mechanical properties, Slow release of essential oils, Mosquito repellent
Plasma Polymerization [102]	Geranium	Resistance to antibiotics	Plasma-induced grafting of essential oil molecules onto surfaces	Durable bonding, Uniform coating, Suitable for sensitive materials
Electrospraying/Electrospinning [103]	Clove, Oregano	Filtration Systems, Wound Dressings	Formation of fibers or particles loaded with essential oils via electrostatic forces	High surface area, Efficient use of essential oils, Applicable to complex shapes
Dip Coating [104]	Eucalyptus, Lavender	Food Packaging, Cutlery	Immersion of the object into an essential oil-infused solution	Antimicrobial properties, Simple implementation, Biodegradability

## Data Availability

Not applicable.
